# Supporting Family Caregiver Health in Heart Failure: Longitudinal Associations Between Heart Failure Caregiver Self-Care and Patient Hospitalizations

**DOI:** 10.1016/j.cardfail.2026.03.023

**Published:** 2026-03-25

**Authors:** JULIE T. BIDWELL, RYAN QUINN, KAREN B. HIRSCHMAN, KATHRYN H. BOWLES, GLADYS L. THOMAS, MICHAEL A. STAWNYCHY, AUSTIN MATUS, JOYCE W. WALD, ALEX J. FAUER, BARBARA RIEGEL

**Affiliations:** 1 Betty Irene Moore School of Nursing, University of California Davis, Davis California;; 2 School of Nursing, University of Pennsylvania, Philadelphia, Pennsylvania;; 3 Center for Home Care Policy & Research at VNS Health, New York, New York; 4 Penn Medicine, Chester County Hospital, West Chester, Pennsylvania.

**Keywords:** Caregivers, heart failure, self-care, hospitalization

## Abstract

**Background:**

Health coaching has been shown to reduce heart failure (HF) caregiver stress and increase caregiver engagement in healthy lifestyle behaviors (“self-care”). The objective of this study was to examine potential downstream effects of improvements in self-care of the caregiver on HF patient hospitalization outcomes.

**Methods and Results:**

Data were from a randomized controlled trial testing the efficacy of a health coaching intervention vs information-only control on HF caregiver self-care. Self-care of the caregiver was measured using the Self-Care Inventory. Care recipient hospitalizations and hospitalization days were abstracted from the medical record. Zero-inflated negative binomial models were used to assess the extent to which caregiver self-care improvement, of any degree and regardless of intervention condition, was predictive of patient hospitalization and hospitalization days, adjusting for common confounders. The sample included 125 HF patient–caregiver dyads from both groups, 61.6% of which were spousal/partnered. Nearly two-thirds of caregivers (62.4%) demonstrated self-care improvement. Approximately one-quarter of patients (25.6%) experienced at least 1 hospitalization. Adjusting for confounders, patients with caregivers who improved in self-care experienced significantly fewer hospitalizations (incidence rate ratio 0.332, 95% confidence interval 0.131–0.841, *P* = .02). No significant effect was observed for hospital days (incidence rate ratio, 0.773, 95% confidence interval 0.271–2.206).

**Conclusions:**

Improvement in HF caregiver self-care was associated with a 67% decrease in patient hospitalizations. Our findings support additional investment in research, programs, and policies that support caregiver health. Our findings may also reassure caregivers, patients, and clinicians that investing time in supporting caregiver health is time well spent and may have potential benefit for patient outcomes.

**Trial registration:**

ClinicalTrials.gov, NCT03988621

## Lay Summary

Family caregivers go by many names—care partners, copatients, cosurvivors, carers—but regardless of their title, we all know that caregivers play a critical role in supporting the care and health of individuals living with heart failure. We also know that many caregivers sometimes put their own health on the back burner when they are juggling the demands of caregiving and everyday life. In this study, we asked the question, “What happens when caregivers make investments in caring for their own health—could that have positive downstream impacts for the person they care for?” To answer this question, we used data from a trial of a health coaching intervention for caregivers of persons living with heart failure. Many trials for heart failure caregivers focus on helping the caregiver to learn how to better support the patient’s health, but this trial was different. It focused entirely on helping the caregiver focus on their own health by improving everyday self-care (ie, health-promoting activities like exercise, healthy eating, preventing illness, and managing stress). In this trial, many caregivers improved their own self-care. So we examined whether a caregiver’s improvement in their own self-care predicted fewer hospitalizations for the person they cared for in the months that followed. And we found that caregiver improvement in self-care—to any degree—was associated with 67% fewer hospitalizations for the person living with heart failure. This finding is important for many reasons, but if you are a caregiver who might be worried that spending time on your own self-care could somehow take away from the health of your loved one with heart failure, we hope that reading our paper will provide reassurance and useful tools to help you invest in your own health amid the challenges of caregiving in everyday life.

Heart failure (HF) is a major source of health care utilization and costs in the United States, with hospitalization being a primary driver.^1^ Average total health care costs for older adults in the year after a HF hospitalization exceeds $40,000, and of greater concern, hospitalization places persons living with HF at higher risk for death.^1,2^ Specifically, 1-year mortality after HF hospitalization is 35.2% for US adults aged 65 and older^2^ and 5-year mortality approaches 80%.^3^ Policy solutions to address the burden of HF hospitalization, such as the Hospital Readmission Reduction Program implemented by the US Centers for Medicaid & Medicare Services, have seen mixed success, with hospitalizations increasing, rather than decreasing, alongside increases in mortality that have exceeded 45%.^4,5^

Policies penalizing HF hospitalizations have also played a role in shifting complex HF care into the home environment by incentivizing systems to deploy home management strategies for exacerbations that would have previously triggered hospitalization.^6^ Family caregivers, defined as family members or fictive kin that help the person with HF with illness management and other health and functional needs, bear the majority of the caregiving burden created by these shifts in care delivery.^6,7^ Caregivers provide support across the spectrum of HF management, as well as support for caregiving activities not specific to HF, such as activities of daily living and care coordination.^7–9^

Although caregiving support is often associated with better outcomes for the person living with HF,^7,9^ caregiving also comes with risks to caregivers, including elevated stress, economic strain, loss of social relationships, and compromised physical and mental health.^7–11^ Caregivers’ ability to sustain their caregiving role is also threatened when they cannot care for their own health due to the high demands of caregiving.^12,13^ Despite this, most HF caregiving interventions focus on providing caregivers with HF-specific education or caregiving skills training, rather than supporting the caregiver’s own health.^9^ To address this intervention gap, members of our group developed the Virtual Caregiver Coach for You (ViCCY) health coaching intervention to support HF caregivers’ engagement in self-care to support their own health, and tested the intervention in a randomized controlled trial (NCT03988621).^14–16^ Self-care refers to the health-promoting behaviors a person engages in to maintain health and prevent or manage illness.^17^ It is an important predictor of physical and mental health status, and, in caregivers, there is evidence that poor self-care can adversely impact caregiver health.^18,19^ Caregivers who received the ViCCY intervention experienced significantly greater improvements in self-care and mental health and decreases in stress as compared with caregivers allocated to an information-only control group.^16^

In the present study, we address an additional a priori aim of the ViCCY intervention study centered on exploring potential effects of self-care of the caregiver on patient clinical outcomes.^15^ Although HF caregiving studies that include patient end points are rare,^7^ HF caregiver characteristics such as better mental health and preparedness for caregiving have been associated with a lower patient risk for hospitalization or mortality.^20,21^ These findings suggest that caregivers’ well-being may have clinically meaningful spillover benefits for patient outcomes,^22,23^ but this has been relatively unexplored in HF.^7,24^ To address this gap, our overall objective was to explore the effect of self-care on the caregiver on HF patient hospitalization outcomes. Specifically, we tested the a priori hypothesis that, at 12 months after caregiver enrollment, HF patients whose caregivers improved in self-care vs those who did not, regardless of study group, would have lower hospitalization rates and fewer hospital days. Our intent in examining patient outcomes in relation to changes in caregiver self-care, rather than the intervention itself, was to generate broader mechanistic insights for future research, practice, and policy.

## Methods

### Study Design and Sample

Data come from a phase II randomized controlled trial testing the efficacy of the ViCCY evidence-based intervention designed to improve self-care among caregivers of persons with HF. Details of the study design and results have been published previously.^15,16^ Between 2019 and 2022, the study consecutively enrolled HF caregivers from acute care and outpatient settings of a major academic health system in the northeastern United States. Caregivers were recruited using in-person and virtual modalities. To be included, caregivers needed to (a) provide at least 8 hours of care per week for a person living with HF, (b) screen positive for poor self-care (score of ≥2 on the Health Self-Care Neglect Scale^25^), and (c) be able to read and speak in English. Caregivers were excluded if (a) they had cognitive impairment or an untreated major psychiatric disorder, (b) they were participating in another supportive intervention study, or (c) the person they cared for was enrolled in hospice. Because dyadic data were required to test the hypotheses in the present study, additional criteria were used to define the subsample. This included enrollment of the person living with HF (consent to completing short surveys and abstraction of clinical data) and that the person living with HF must be medically managed (could not be a recipient of advanced therapies, such as mechanical circulatory support or a transplant). The decision to focus on dyads with medically managed patients was due to known differences in caregiving experiences and patient health care utilization between those who are medically managed vs those receiving advanced therapies.^7,26^ Of the 250 caregivers enrolled, 173 were caring for a person with medically managed HF, and of those, 125 had complete dyadic data for analysis. The study’s protocol was approved by the University of Pennsylvania’s Institutional Review Board, and all participants gave informed consent.

### Intervention and Control Conditions

Details of the intervention and control conditions have been published previously.^14,15^ Both the intervention and the information-only control conditions were 6 months in duration with outcomes measured at 6 and 12 months. Caregivers in both groups received a tablet device with mobile connectivity, preloaded with curated websites that contained educational content focusing on HF, self-care, and caregiving. All caregivers were instructed to spend at least 30 minutes each week browsing the educational sites of their choice and received monthly reminders to do so. Caregivers allocated to the intervention condition also received personalized 1:1 health coaching delivered virtually over 10 sessions.

### Data Collection

Self-report data was collected from caregivers and patients by a web survey (REDCap).^27^ Patient clinical characteristics and events were abstracted from the medical record. Baseline sociodemographic and clinical data were collected at enrollment. Data on the exposure (caregiver self-care improvement) were collected between enrollment and 6 months post randomization. Data on the outcome (patient hospitalizations) were collected in the 6 months after intervention completion (between 6 and 12 months post randomization). Data collection windows were specified a priori to ensure temporal precedence of the exposure in relation to the outcome.

### Measurement

#### Outcome: Patient Hospitalizations.

Patient hospitalization events within 6 and 12 after following caregiver enrollment were operationalized in 2 ways: all-cause hospitalizations (count) and hospital days (count). Hospitalization data were abstracted from the patient’s medical record by trained research assistants blinded to group allocation. All hospitalization events were verified with participants during follow-up calls, and participants were additionally asked about any hospitalizations occurring outside the health system that may have been missed.

#### Exposure: Improvement in Self-Care of the Caregiver.

Caregiver improvement in self-care, regardless of intervention group, was operationalized as a dichotomous variable representing any level of self-care improvement between enrollment and 6 months post randomization (change in score of >0) vs no improvement or worsening (change in score of ≤0). Self-care was measured using the Maintenance scale of the Self-Care Inventory,^28^ which consists of 8 items asking respondents how often they do the following health-promoting self-care behaviors: sufficient sleep, illness prevention (handwashing, vaccination), physical activity, healthy diet, routine preventive care, taking medicines as prescribed, stress management (eg, meditation, yoga), and minimizing tobacco smoke exposure. Although the Self-Care Inventory has 3 scales—Maintenance, Monitoring, and Management—the Maintenance scale was selected because it directly reflects the content delivered in the ViCCY intervention. The Maintenance scale of the Self-Care Inventory has established evidence of validity and reliability in a general adult population (global reliability index of 0.85, test retest–reliability of 0.81).^28^ Responses are rated on a 5-point Likert scale from 1 (never) to 5 (always). Responses are summed across items, then normalized to range from 0 to 100, with higher scores indicating better self-care for the caregiver. Although there is no established minimally important difference for change in self-care, the instrument developers recommend using the convention of one-half a standard deviation in baseline scores, or (in lieu of baseline data) a change of 8 or more points, to evaluate whether a change in self-care is clinically relevant.^29^ In the present study, however, any level of change (rather than specifying a cutoff for clinically meaningful change) was chosen as the exposure variable in accordance with the exploratory nature of our a priori hypothesis.

#### Covariates.

All models were adjusted for demographic and health-related factors that are known confounders of clinical event risk in HF. We adjusted for patient age and sex (male), which are included in almost all HF risk models given that both older age and male sex are associated with higher rates of hospitalization.^1^ We also adjusted for self-identified race (Black or African American), given known disparities in HF clinical outcomes by race/ethnicity, with Black patients experiencing the highest HF-associated hospitalization rates in the United States.^1^ Although there are many underlying drivers of this disparity, including social and structural determinants of health and poorer access to specialty care, limitations on data collected from patients in this study precluded our ability to directly control for those factors.^30,31^ In terms of patient health, we controlled for comorbidities as measured by the Charlson Comorbidity Index.^32^ Comorbidity is commonly included in HF risk models, including our prior study of caregiver-related predictors of patient event risk, given that multimorbidity is highly predictive of poorer outcomes in HF, particularly hospitalization.^33^ Finally, we controlled for caregiving relationship type, given substantial observed differences in outcomes (for both HF patients and caregivers) in prior studies, depending on whether caregiving is occurring within the context of a partnered relationship (eg, spouse or romantic partner) vs some other relationship type (eg, adult child, fictive kin).^9^ We did not control for intervention group, because our a priori objective was to examine effects related to self-care improvement, regardless of whether self-care improved owing to the intervention. However, we did include models examining the effects of the intervention group on patient hospitalization outcomes as part of an additional sensitivity analysis.

### Analysis

Summary statistics were computed using means and standard deviations for continuous measures, and frequencies and percentages for categorical measures. Participant characteristics were compared by caregiver self-care improvement group (care dyads in which the caregiver improved in self-care vs not) using *χ*^2^ tests and 2-sample *t* tests. Zero-inflated negative binomial models were constructed to assess the extent to which self-care improvement was predictive of counts of patient hospitalizations (model 1) and hospital days (model 2). The analysis only focused on hospitalization events from 6 to 12 months to appropriately account for the temporality of the exposure (self-care improvement), which was measured between baseline and the 6-month study timepoint. First, unadjusted models were constructed to assess the effect of self-care improvement without consideration for covariates. Next, adjusted models were constructed in which patient age, sex, race (African American), relationship to caregiver (partnered relationship vs other), and Charlson comorbidity score. Given the count distribution and zero-inflated nature of hospitalization outcomes, model fit was assessed using Akaike and Bayesian information criteria, comparing Poisson and negative binomial specifications as well as their zero-inflated counterparts. The zero-inflated negative binomial specification provided the best fit and was used for the final analysis.

A number of patients (*n* = 13) died during the follow-up period. To minimize selection bias, these patients were not excluded from the analysis. The trajectory of heart failure (HF) in the time period preceding death typically includes recurrent decompensations requiring hospitalization. As such, excluding those who had died would have biased our findings toward a healthier sample of patients, and it would also have removed hospitalizations from the analysis that should have been reasonably included. We also conducted 2 sensitivity analyses: one set of models examining the effects of the intervention group (rather than improvement in caregiver self-care) on patient outcomes, and another set of models examining the effects of caregiver improvement in self-care on patient outcomes in the full study sample (ie, including dyads with patients who had received advanced therapies). Statistical analyses were conducted using SAS version 9.4 for Windows. An alpha level of 0.05 was used to determine statistical significance.

## Results

### Caregiver and Care Recipient Characteristics

Characteristics of patients and caregivers within the 125 dyads included in the present study are presented in Table 1. Patients were in their late 50s on average (59.2 ± 16.8 years), just under one-half (40.8%) were female, and nearly one-third identified as Black or African American (30.6%). In terms of HF classification by ejection fraction, the sample was relatively evenly balanced, with 46.0% of patients having a reduced ejection fraction, 8.1% with mildly reduced ejection fraction, and 46.0% with preserved ejection fraction. In terms of HF functional classification, most patients were New York Heart Association functional class II (41.1%) or III (29.8%). At least 1 hospitalization was observed for 26.4% of patients, with a median hospitalization count of 0 (interquartile range, 0–1) and median hospitalization days count of 0 (interquartile range, 0–0). Histograms showing the distributions of patient hospitalizations and hospital days by caregiver self-care improvement category are provided in the Supplementary Information.

Caregivers were slightly younger than patients on average (56.2 ± 13.1 years), the majority were female (81.6%) and just over one-quarter (27.2%) identified as Black or African American. A slight majority of caregivers were the spouse/partner of the patient (61.6%), with the remainder being a parent or grandparent (16.0%), adult child (12.8%), or other family member or fictive kin (9.6%). On average, caregivers’ baseline score for self-care maintenance was 70.3 ± 14.1. Just over one-half of the dyads (*n* = 78 [62.4%]) included caregivers that exhibited improvement in self-care maintenance from baseline to the 6-month study timepoint, with an average baseline score on the Self-Care Inventory of 65.3 ± 13.4 and an average improvement of 14.5 ± 10.5 points. A histogram showing the distribution of change in caregiver self-care is provided in the Supplementary Material. For context, using the recommended half a standard deviation in baseline scores to evaluate clinical relevance,^29^ a change of 7.05 or more points would be considered clinically meaningful. For those who did not improve (*n* = 47 [37.6%]), the average baseline Self-Care Inventory scores were higher (78.7 ± 11.0) and decreased by 6.9 ± 7.4 points on average.

### Model Results

In unadjusted models, improvement in self-care of the caregiver was not significantly associated with patient hospitalizations (incidence rate ratio [IRR] 0.361, 95% confidence interval [CI] 0.121–1.078, *P* = .068) or hospitalization days (IRR 0.607, 95% CI 0.204–1.808, *P* = .370). After adjusting for patient age, sex, relationship, race, and comorbidity score, a significant effect was observed for self-care improvement for the hospitalization count outcome, such that patients of caregivers who improved in self-care were estimated to experience a 67% decrease in hospitalizations (IRR 0.332, 95% CI 0.131–0.841, *P* = .020) as compared with patients of caregivers who did not improve in self-care (Table 2). In the adjusted model for hospitalization days, a significant effect for self-care maintenance improvement was not observed (IRR 0.773, 95% CI 0.271–2.206, *P* = .630) (Table 3). In our sensitivity analyses examining the effects of the intervention group rather than improvement in caregiver self-care, the intervention group was not significantly associated with either the count of hospitalizations or the count of hospital days; however, it was significantly associated with higher odds of patients having zero hospitalizations and zero hospital days (IRR 5.237, 95% CI 1.173–23.373, *P* = .030 and IRR 2.947, 95% CI 1.108–7.839, *P* = .030, respectively). In our sensitivity analyses with the full sample (ie, including dyads with patients who had received advanced therapies), self-care improvement was not significantly associated with either hospitalizations or hospital days. Full models including the reporting of estimates for the zero components, negative binomial dispersion (scale) parameters, and all sensitivity analyses are provided in the Supplementary Material.

## Discussion

In this study, improvement in caregiver self-care, regardless of intervention grouping, was significantly associated with fewer all-cause hospitalizations for patients, but not length of stay (all-cause hospital days). Relatively few studies in HF have examined the impact of caregiver characteristics on clinical events or health care utilization outcomes for the person living with HF, but what is known suggests that better caregiver health—specifically mental health—is associated with better patient clinical outcomes, including hospitalization and rehospitalization, mortality, and event-free survival.^7,23^ Although these findings suggest that supporting caregivers with their own health could have downstream clinical benefits for patients, the majority of HF caregiving interventions focus instead on providing caregivers with HF-specific education and improving their caregiving skills and contributions.^9^ These interventions also report primarily on caregiver outcomes, with limited or no data on patient outcomes.^9^ As such, both our intervention’s focus on caregiver health and our study’s examination of potential downstream effects of improving self-care of the caregiver on patient clinical outcomes represents novel contributions in HF.

In the broader caregiving literature, interventions that share commonalities with our health coaching intervention have shown positive effects on patient health care utilization outcomes. For example, the REACH and COPE interventions were designed to support the health of caregivers and care dyads living with dementia, and both have been associated with lower health care costs.^34,35^ In COPE, these decreases were driven by lower utilization of acute care (fewer hospitalizations and emergency visits) and nursing home care.^35^ A fundamental principle of these interventions is that they are tailored to the individual caregiver, with both including comprehensive assessments that identify areas of caregiver need that are then used to guide personalized support. This principle is shared by our health coaching intervention, which begins with a caregiver assessment that centers on caregiver needs and drives goal setting and action planning with the health coach for the duration of the intervention.^15^

Although our intervention includes key principles of other effective interventions, our a priori aim was not to examine the direct effect of the intervention, but rather to examine whether improvement in self-care of the caregiver, regardless of intervention grouping, was associated with subsequent patient outcomes. Our finding that caregiver self-care improvement, of any degree, was associated with a decrease in patient hospitalizations warrants exploration of potential mechanisms. One possibility is that improving self-care bolsters caregiver mental and physical health, thereby improving caregivers’ capacity to meet patients’ care needs. Caregivers who appraise caregiving as emotionally and physically difficult may be less able to meet patients’ care needs,^36^ and unmet needs have been associated with adverse patient outcomes, including higher health care utilization.^37^ Self-care improvement may also make caregiving activities more sustainable overall, thus translating to better patient outcomes over time. Another possibility is that caregiver improvements in their own self-care may positively influence the patient’s own health behaviors. In HF care dyads, many studies have demonstrated significant interdependencies in patient and caregiver health, as well as important transactional effects.^9^ For example, better mental (and relational) health in caregivers has been found to predict higher patient self-efficacy to care for their own HF.^38^

In terms of research implications, focusing on the effects of self-care improvement on patient outcomes, rather than the intervention itself, provides researchers with important future directions. For observational researchers, this finding is hypothesis generating, because it provides the rationale for a closer examination of caregiver health and real-world engagement in health-promoting activities as potential upstream mechanisms of both caregiver and patient outcomes. For interventionists, our findings provide the scientific premise for further testing and potential implementation of tailorable interventions that target caregiver health and self-care, either in lieu of or in combination with traditional caregiving intervention components that provide caregiving-specific education, skills, and linkages to services. Our study also provides justification for more consistent collection of data on patient outcomes. Although collecting patient data adds cost and complexity to caregiving studies, the lack of patient outcomes data—particularly clinical outcomes and health care utilization data—persists as a field-limiting research gap.

Limited data demonstrating caregivers’ downstream impacts on patient outcomes has also made it difficult to justify health care system integration and reimbursement of supportive caregiving services. As such, our findings have implications for policy implementation. Not only was the ViCCY intervention efficacious in improving self-care of the caregiver and reducing caregiver stress,^16^ but the associations with reduced patient hospitalization in our study suggest that services aimed at supporting caregiver health and self-care (eg, health coaching or similar caregiver-focused health promotion interventions) may be valuable to improve outcomes for the person living with HF. Interventions and services should also fully consider caregiver social and structural determinants of health and address unmet health-related social needs and gaps in access to services.^22,39,40^ Assessment for unmet caregiver needs and associated referrals for practical services and supports could feasibly be integrated into health coaching programs, further supporting caregivers’ ability to prioritize their own health. Interventions that link caregivers to practical resources are critical to ensuring they have the support needed to engage in their own self-care, as coaching focused on health alone is unlikely to address underlying social determinants of health. To implement and reap the benefits of such services, however, caregiving-focused policies and programs must be prioritized. In the United States, policy and programmatic efforts to implement the 2022 National Strategy to Support Family Caregivers must continue, and momentum in recent years to expand Medicare and Medicaid services that support caregivers, alongside payment mechanisms for caregiver assessment and support in the US Centers for Medicaid & Medicare Services Physician Fee Schedule, must not be lost.^41^ We must also preserve key federal programs that provide grants to states to implement critical supportive services to caregivers, such as the National Family Caregiver Support Program and others funded through the Administration of Community Living.

Our study also has implications for practice. Clinical guidelines for HF call for the inclusion and support of HF caregivers as part of clinical care, but provide limited guidance on how that support should be provided.^42^ Our study adds to the growing body of HF caregiving knowledge that offers clinicians evidence-based options for supporting HF caregivers. This study also provides clinicians with much-needed evidence to use in discussions with caregivers about the value of caring for their own health. Because many caregivers neglect their own health out of concern for the health of the patient,^12,13^ our findings can be used to counter that concern and reassure caregivers that investing in their own self-care may potentially benefit, rather than harm, the health of the care recipient. For caregivers who want to improve their self-care but struggle to identify effective strategies that work amid the challenges of everyday life, several organizations known for their advocacy and support of family caregivers, such as the Family Caregiver Alliance and AARP, have compiled resources related to self-care and health for caregivers. Caregivers may also be interested in exploring the Powerful Tools for Caregivers program, which is an evidence-based set of tools focused on improving caregivers’ self-care and their ability to handle difficult caregiving-related situations.^25^

Our findings must be considered in the context of certain limitations. First, the study was conducted at a single center in the United States and enrolled only caregivers with existing self-care deficits and English proficiency. Additionally, as an exploratory aim, no a priori power analyses were conducted; as such, it is difficult to disentangle whether our lack of findings related to hospitalization days represents true null findings or a lack of power. The relatively small number of hospitalizations and hospital days experienced by patients in the study is also a limitation, and points to a need for future work in larger, more representative samples. Furthermore, to minimize barriers to patient participation and reduce their survey burden, we have limited data on patient-reported characteristics or person-centered outcomes. Although limiting the length of the patient survey likely supported higher patient enrollment and survey completion, it also created a tradeoff in our ability to quantify and adjust for the complex influences on patient hospitalization outcomes, particularly social and structural determinants of health. We were also not able to consistently abstract certain clinical variables that are often included in HF event-risk modeling, such as prior health care utilization. Additionally, although we found a significant effect for patient hospitalizations, we found no such effect for hospitalization days. The drivers of hospitalization and length of stay are often different and require more nuanced data to disentangle than we had available in the present study.^43^ In future studies, additional data on patient clinical characteristics, disease severity, and details of the hospitalization (eg, planned or unplanned, secondary diagnoses, adverse hospital events) may shed light on this finding.

This study also has strengths, including success in recruiting a more racially diverse sample than is typical in studies of HF care dyads^9^ and a design that accounted for the temporal precedence of exposure and outcome. Our ability to collect longitudinal outcomes on HF patients is a notable strength as many studies that examine linkages between caregiver characteristics and HF patient outcomes are overwhelmingly cross-sectional or have shorter follow-up periods.^7,9^ We were also able to collect and adjudicate clinical outcomes data from patients and examine those outcomes in relation to caregiver outcomes, which is rare and helps to address an important research gap.

## Conclusions

We found that improvement in self-care of the caregiver was significantly associated with a 67% decrease in all-cause hospitalizations for the care recipient living with HF. Our findings represent a novel contribution, as few HF caregiving interventions focus on the caregiver’s own health and self-care, and even fewer examine downstream patient clinical outcomes. Important next steps for research include further examination of the mechanisms by which caregiver health supports patient outcomes, a special focus on the role of social and structural determinants of health for both patient and caregiver health, and further testing and implementation of interventions and services that support caregiver health and the promotion of sustainable, healthy caregiving. Alongside decades of caregiving research across conditions, our findings also add to the body of knowledge that unequivocally demonstrates the value of continued and expanded investments in health policies and programs that support caregivers. Our study also provides evidentiary support for important clinical conversations with caregivers who may be neglecting their own health out of concerns that taking time to care for themselves may adversely impact the patient’s health.

## Supplementary Material

1

Supplementary material associated with this article can be found in the online version at doi:10.1016/j.cardfail.2026.03.023.

## Figures and Tables

**Table 1:** Characteristics of the Sample (N=125 Patient-Caregiver Dyads)

	Full Sample	Caregiver Did Not Improve in Self-Care	Caregiver Improved in Self-Care	
	N=125	n=47(37.6%)	n=78(62.4%)	
	Mean±SD	Mean±SD	Mean±SD	
	or n (%)	or n(%)	or n(%)	p-value
***Patient Characteristics***				
Age (range 20–89 years)	59.2±16.8	62.1 ±16.6	57.5±16.7	0.138
Female Sex	51(40.8)	23(48.9)	28(35.9)	0.189
Race/Ethnicity				
*Black or African-American*	38(30.6)	18(39.1)	20(25.6)	0.055
*White*	76(61.3)	26(56.5)	50(64.1)	
*Hispanic or Latino*	1(0.8)	0(0.0)	1(1.3)	
*Other or More Than One Race*	9(7.3)	2(4.4)	7(9.0)	
NYHA Functional Classification				
*NYHA Class I*	14(11.3)	6(12.8)	8(10.4)	0.498
*NYHA Class II*	51(41.1)	19(40.4)	32(41.6)	
*NYHA Class III*	37(29.8)	17(36.2)	20(26.0)	
*NYHA Class IV*	11(8.9)	3(6.4)	8(10.4)	
*Not available*	11(8.9)	2(4.3)	9(11.7)	
Left Ventricular Ejection Fraction (LVEF; range 10–85)	41.1 ±20.1	41.9±21.7	40.6±19.2	0.719
Heart Failure Classification by Ejection Fraction				
*HFrEF (LVEF* ≤*40%)*	57(46.0)	21(44.7)	36(46.8)	0.886
*HFmrEF (LVEF 41–49%)*	10(8.1)	3(6.4)	7(9.1)	
*HFpEF (LVEF* ≥*50%)*	57(46.0)	23(48.9)	34(44.2)	
Charlson Comorbidity Score (range 1–9)	3.1±2.0	3.1±1.9	3.2±2.0	0.781
Hospitalized During Follow-Up	32(25.6)	12(25.5)	20(25.6)	1.000
Hospitalization Count Mean (range 0–9)	0.5±1.27	0.7±1.6	0.4±1.0	0.289
Hospitalization Count Median (Q1, Q3)	0 (0, 0.5)	0(0, 0)	0(0, 1)	
Hospital Days Mean (range 0–70)	3.6±11.1	4.9±14.5	2.8±8.3	0.310
Hospital Days Median (Q1, Q3)	0(0, 0)	0(0, 0)	0(0, 0)	
***Caregiving Relationship Type (Caregiver's Relationship to Patient)***				
Spouse/Partner	77(61.6)	25(53.2)	52(66.7)	0.220
Child/Child-in-law	16(12.8)	5(10.6)	11(14.1)	
Parent/Grandparent	20(16.0)	11(23.4)	9(11.5)	
Other	12(9.6)	6(12.8)	6(7.7)	
***Caregiver Characteristics***				
Age (range 19–77 years)	56.2±13.1	56.7±13.0	55.8±13.2	0.708
Female Gender	102(81.6)	39(83.0)	63(80.8)	0.816
Race/Ethnicity				
*Black or African-American*	34(27.2)	18(38.3)	16(20.5)	0.076
*White*	80(64.0)	26(55.3)	54(69.2)	
*Hispanic or Latino*	1(0.8)	1(2.1)	0(0.0)	
*Other or More Than One Race*	10(8.0)	2(4.3)	8(10.3)	
Educational Attainment				
*High School or Less*	27(21.8)	8(17.0)	19(24.7)	0.263
*Trade School or Some College*	43(34.7)	14(29.8)	29(37.7)	
*Bachelor's degree or more*	54(43.5)	25(53.2)	29(37.7)	
Unmet Health-Related Social Needs				
*0*	100(80.0)	38(80.9)	62(79.5)	0.717
*1*	16(12.8)	5(10.6)	11(14.1)	
*2*	4(3.2)	1(2.1)	3(3.8)	
*3 or more*	5(4.0)	3(6.4)	2(2.6)	
Income insufficient to make ends meet	18(15.0)	5(11.6)	13(16.9)	0.596
Quit or took early retirement to provide caregiving support	25(20.0)	9(19.1)	16(20.5)	1.000
Years Caregiving (range 0–45)	6.6±8.3	5.3±6.4	7.4±9.3	0.174
Hours Per Day Caregiving (range 1–24)	7.3±6.9	6.7±6.2	7.7±7.1	0.435
Self-Care Maintenance (Baseline, range 25–97)	70.3±14.1	78.7±11.0	65.3±13.4	>0.001
Self-Care Maintenance (6 Months, range 47–100)	76.7±12.7	71.7±12.7	79.8±11.8	0.001
Change in Self-Care (Baseline to 6 Months, range −34.4 – 50.0)	6.4±14.0	−6.9±7.4	14.5±10.5	>0.001

Notes: NYHA: New York Heart Association; LVEF: left ventricular ejection fraction; HFrEF: heart failure with reduced ejection fraction; HFmrEF: heart failure with mildly reduced ejection fraction; HFpEF: heart failure with preserved ejection fraction; Unmet social needs: count of how many health-related social needs (food, clothing, utilities, childcare, medicine/healthcare, phone, or other) the respondent has been unable to obtain in the past year, measured using a modified version of the PRAPARE tool.

**Table 2:** Fully Adjusted Model Predicting the Patient All-Cause Hospitalizations (Count) in the 6–12 months After Intervention Completion

	IRR	95% Confidence Interval	*p*-value
Caregiver Improved in Self-Care	0.332	[0.131,0.841]	0.020
Patient Charlson Comorbidity Score	1.103	[0.889,1.368]	0.374
Spousal/Partnered Relationship	1.274	[0.456,3.557]	0.644
Patient Age	0.952	[0.926,0.980]	<0.001
Patient Sex (Female)	0.371	[0.139,0.993]	0.048
Patient Race (Black or African American)	1.746	[0.608,5.012]	0.300

Notes: Negative binomial regression model, zero components not shown (all non-significant); outcome is patient all-cause hospitalizations (count) in the 6–12 months after the patient’s caregiver completed the intervention or attention-control condition; IRR: Incidence rate ratio.

**Table 3:** Fully Adjusted Model Predicting the Patient All-Cause Hospitalization Days (Count) in the 6–12 months After Intervention Completion

	IRR	Interval	*p*-value
Caregiver Improved in Self-Care	0.773	[0.271,2.206]	0.630
Patient Charlson Comorbidity Score	1.074	[0.860,1.340]	0.529
Spousal/Partnered Relationship	0.661	[0.202,2.161]	0.493
Patient Age	0.946	[0.914,0.979]	0.002
Patient Sex (Female)	0.717	[0.219,2.352]	0.583
Patient Race (Black or African American)	1.485	[0.440,5.007]	0.524

Notes: Negative binomial regression model, zero components not shown (all non-significant with the exception of the Charlson score); outcome is the patient all-cause hospitalization days (count) in the 6–12 months after the patient’s caregiver completed the intervention or attention-control condition; IRR: Incidence rate ratio.
