# Supplementary material for: Supporting Family Caregiver Health in Heart Failure: Longitudinal Associations Between Heart Failure Caregiver Self-Care and Patient Hospitalizations
Source: J Card Fail. Author manuscript; Available in PMC 2026 Jul 13. (PMC13359039; doi:10.1016/j.cardfail.2026.03.023)
Supplement: 1 [file NIHMS2191446-supplement-1.docx]

**SUPPLEMENT**

**Full negative binomial regression models for final analyses including zero components, with estimates reported in the original metric (log scale). In the first set of models (A), the outcome is patient all-cause hospitalizations (count) in the 6-12 months after the patient’s caregiver completed the intervention or attention-control condition. In the second set of models (B), the outcome is patient all-cause hospitalization days (count), also in the 6-12 months after the patient’s caregiver completed the intervention or attention-control condition.**

| **A. Models Predicting Hospitalization Count** | | | | | | | | | | |
| --- | --- | --- | --- | --- | --- | --- | --- | --- | --- | --- |
|  | **Unadjusted** | | | | | | | | | |
|  | **Poisson Component** | | | | | **Zero Component** | | | | |
| **Predictors** | Estimate | Standard Error | Lower | Upper | p | Estimate | Standard Error | Lower | Upper | p |
| Caregiver Improved in Self-Care | -1.017 | 0.557 | -2.109 | 0.075 | 0.068 | -22.540 | 84546.1 | -165730 | 165684.8 | 0.999 |
| Dispersion | 2.1226 | 0.9858 | 0.8542 | 5.2744 |  |  |  |  |  |  |
|  | **Adjusted** | | | | | | | | | |
|  | **Poisson Component** | | | | | **Zero Component** | | | | |
|  | Estimate | Standard Error | Lower | Upper | p | Estimate | Standard Error | Lower | Upper | p |
| Caregiver Improved in Self-Care | -1.104 | 0.475 | -2.035 | -0.174 | 0.020 | -4.632 | 3.772 | -12.024 | 2.761 | 0.220 |
| Patient Charlson Score | 0.098 | 0.110 | -0.118 | 0.314 | 0.374 | -1.190 | 0.933 | -3.019 | 0.639 | 0.202 |
| Patient Sex (Female) | -0.990 | 0.502 | -1.974 | -0.007 | 0.048 | -3.472 | 2.333 | -8.044 | 1.100 | 0.137 |
| Patient Race (Black or African American) | 0.557 | 0.538 | -0.497 | 1.612 | 0.300 | -1.940 | 2.141 | -6.136 | 2.257 | 0.365 |
| Spousal/Partnered Relationship | 0.242 | 0.524 | -0.785 | 1.269 | 0.644 | -0.366 | 1.851 | -3.994 | 3.263 | 0.844 |
| Age | -0.049 | 0.014 | -0.077 | -0.021 | 0.001 | -0.110 | 0.096 | -0.298 | 0.077 | 0.250 |
| Dispersion | 0.8396 | 0.6016 | 0.2061 | 3.4199 |  |  |  |  |  |  |
| **B. Models Predicting Hospitalization Days** | | | | | | | | | | |
|  | **Unadjusted** | | | | | | | | | |
|  | **Poisson Component** | | | | | **Zero Component** | | | | |
| **Predictors** | Estimate | Standard Error | Lower | Upper | p | Estimate | Standard Error | Lower | Upper | p |
| Caregiver Improved in Self-Care | -0.499 | 0.557 | -1.590 | 0.592 | 0.370 | 0.079 | 0.488 | -0.877 | 1.035 | 0.871 |
| Dispersion | 2.5081 | 1.5025 | 0.7752 | 8.1144 |  |  |  |  |  |  |
|  | **Adjusted** | | | | | | | | | |
|  | **Poisson Component** | | | | | **Zero Component** | | | | |
|  | Estimate | Standard Error | Lower | Upper | p | Estimate | Standard Error | Lower | Upper | p |
| Caregiver Improved in Self-Care | -0.257 | 0.535 | -1.306 | 0.791 | 0.630 | 0.435 | 0.512 | -0.568 | 1.439 | 0.395 |
| Patient Charlson Score | 0.071 | 0.113 | -0.150 | 0.293 | 0.529 | -0.321 | 0.126 | -0.567 | -0.075 | 0.011 |
| Patient Sex (Female) | -0.333 | 0.606 | -1.521 | 0.855 | 0.583 | 0.266 | 0.541 | -0.794 | 1.325 | 0.623 |
| Patient Race (Black or African American) | 0.395 | 0.620 | -0.821 | 1.611 | 0.524 | -0.135 | 0.551 | -1.215 | 0.946 | 0.807 |
| Spousal/Partnered Relationship | -0.414 | 0.604 | -1.598 | 0.770 | 0.493 | -0.823 | 0.566 | -1.933 | 0.287 | 0.146 |
| Age | -0.056 | 0.018 | -0.090 | -0.021 | 0.002 | 0.016 | 0.017 | -0.018 | 0.050 | 0.348 |
| Dispersion | 1.0645 | 0.4457 | 0.4686 | 2.4184 |  |  |  |  |  |  |

**Sensitivity analyses examining the effects of the intervention group rather than improvement in caregiver self-care: Full negative binomial regression models including zero components, with estimates reported in the original metric (log scale). In the first model (A), the outcome is patient all-cause hospitalizations (count) in the 6-12 months after the patient’s caregiver completed the intervention or attention-control condition. In the second model (B), the outcome is patient all-cause hospitalization days (count), also in the 6-12 months after the patient’s caregiver completed the intervention or attention-control condition.**

| **A. Model Predicting Hospitalization Count** | | | | | | | | | | |
| --- | --- | --- | --- | --- | --- | --- | --- | --- | --- | --- |
|  | **Poisson Component** | | | | | **Zero Component** | | | | |
|  | Estimate | Standard Error | Lower | Upper | p | Estimate | Standard Error | Lower | Upper | p |
| Caregiver Randomized to Intervention | 0.600 | 0.418 | -0.220 | 1.420 | 0.152 | 1.656 | 0.763 | 0.160 | 3.152 | 0.030 |
| Patient Charlson Score | 0.082 | 0.109 | -0.132 | 0.295 | 0.454 | -0.291 | 0.182 | -0.648 | 0.066 | 0.111 |
| Patient Sex (Female) | 0.096 | 0.464 | -0.813 | 1.005 | 0.836 | 0.681 | 0.960 | -1.200 | 2.562 | 0.478 |
| Patient Race (Black or African American) | 1.150 | 0.445 | 0.278 | 2.021 | 0.010 | 0.575 | 0.856 | -1.104 | 2.254 | 0.502 |
| Spousal/Partnered Relationship | 0.164 | 0.461 | -0.739 | 1.067 | 0.722 | -0.431 | 0.890 | -2.176 | 1.314 | 0.629 |
| Age | -0.020 | 0.015 | -0.048 | 0.009 | 0.175 | 0.009 | 0.025 | -0.039 | 0.058 | 0.707 |
| Dispersion | 0.0533 | 0.4339 | 0.0 | 455597.6 |  |  |  |  |  |  |
| **B. Model Predicting Hospitalization Days** | | | | | | | | | | |
|  | **Poisson Component** | | | | | **Zero Component** | | | | |
|  | Estimate | Standard Error | Lower | Upper | P | Estimate | Standard Error | Lower | Upper | P |
| Caregiver Randomized to Intervention | 0.382 | 0.451 | -0.502 | 1.267 | 0.397 | 1.081 | 0.499 | 0.103 | 2.059 | 0.030 |
| Patient Charlson Score | 0.067 | 0.106 | -0.140 | 0.274 | 0.528 | -0.294 | 0.122 | -0.533 | -0.055 | 0.016 |
| Patient Sex (Female) | -0.060 | 0.503 | -1.045 | 0.925 | 0.905 | 0.243 | 0.537 | -0.810 | 1.295 | 0.651 |
| Patient Race (Black or African American) | 0.332 | 0.610 | -0.864 | 1.528 | 0.586 | -0.246 | 0.563 | -1.350 | 0.858 | 0.662 |
| Spousal/Partnered Relationship | -0.450 | 0.599 | -1.623 | 0.724 | 0.453 | -0.854 | 0.575 | -1.982 | 0.274 | 0.138 |
| Age | -0.051 | 0.018 | -0.086 | -0.017 | 0.004 | 0.015 | 0.017 | -0.018 | 0.049 | 0.365 |
| Dispersion | 1.0024 | 0.3995 | 0.4590 | 2.1892 |  |  |  |  |  |  |

**Sensitivity analyses examining the effects of caregiver self-care improvement in the full sample (i.e., including dyads with patients who had received advanced therapies, N=174 with complete data for analysis): Full negative binomial regression models including zero components, with estimates reported in the original metric (log scale). In the first model (A), the outcome is patient all-cause hospitalizations (count) in the 6-12 months after the patient’s caregiver completed the intervention or attention-control condition. In the second model (B), the outcome is patient all-cause hospitalization days (count), also in the 6-12 months after the patient’s caregiver completed the intervention or attention-control condition.**

| **A. Model Predicting Hospitalization Count** | | | | | | | | | | |
| --- | --- | --- | --- | --- | --- | --- | --- | --- | --- | --- |
|  | **Poisson Component** | | | | | **Zero Component** | | | | |
|  | Estimate | Standard Error | Lower | Upper | p | Estimate | Standard Error | Lower | Upper | p |
| Caregiver Improved in Self-Care | -0.630 | 0.392 | -1.398 | 0.138 | 0.108 | -0.320 | 0.646 | -1.586 | 0.946 | 0.620 |
| Patient Charlson Score | -0.028 | 0.101 | -0.225 | 0.169 | 0.781 | -0.228 | 0.198 | -0.616 | 0.160 | 0.249 |
| Patient Sex (Female) | -0.154 | 0.494 | -1.122 | 0.814 | 0.755 | 0.042 | 0.849 | -1.623 | 1.707 | 0.961 |
| Patient Race (Black or African American) | 1.254 | 0.401 | 0.469 | 2.039 | 0.002 | 0.645 | 0.725 | -0.776 | 2.067 | 0.373 |
| Spousal/Partnered Relationship | 0.373 | 0.456 | -0.520 | 1.267 | 0.413 | 0.075 | 0.861 | -1.613 | 1.763 | 0.931 |
| Age | -0.039 | 0.015 | -0.068 | -0.011 | 0.007 | -0.024 | 0.029 | -0.080 | 0.032 | 0.406 |
| Scale | 1.00 | 0.00 | 1.00 | 1.00 |  |  |  |  |  |  |
| **B. Model Predicting Hospitalization Days** | | | | | | | | | | |
|  | **Poisson Component** | | | | | **Zero Component** | | | | |
|  | Estimate | Standard Error | Lower | Upper | P | Estimate | Standard Error | Lower | Upper | P |
| Caregiver Improved in Self-Care | 0.096 | 0.396 | -0.679 | 0.871 | 0.808 | 0.302 | 0.414 | -0.510 | 1.114 | 0.466 |
| Patient Charlson Score | -0.010 | 0.103 | -0.212 | 0.192 | 0.922 | -0.142 | 0.097 | -0.332 | 0.047 | 0.142 |
| Patient Sex (Female) | 0.380 | 0.458 | -0.518 | 1.278 | 0.407 | 0.278 | 0.449 | -0.601 | 1.157 | 0.535 |
| Patient Race (Black or African American) | 0.335 | 0.534 | -0.713 | 1.382 | 0.531 | -0.402 | 0.439 | -1.263 | 0.459 | 0.361 |
| Spousal/Partnered Relationship | -0.240 | 0.574 | -1.364 | 0.885 | 0.676 | -0.443 | 0.466 | -1.357 | 0.471 | 0.343 |
| Age | -0.044 | 0.017 | -0.078 | -0.010 | 0.012 | 0.009 | 0.013 | -0.017 | 0.033 | 0.514 |
| Dispersion | 1.1285 | 0.4086 | 0.5550 | 2.2946 |  |  |  |  |  |  |

**Histogram Showing the Distribution of Caregiver Self-Care Change Scores Between Enrollment and 6 Months Post-Randomization**


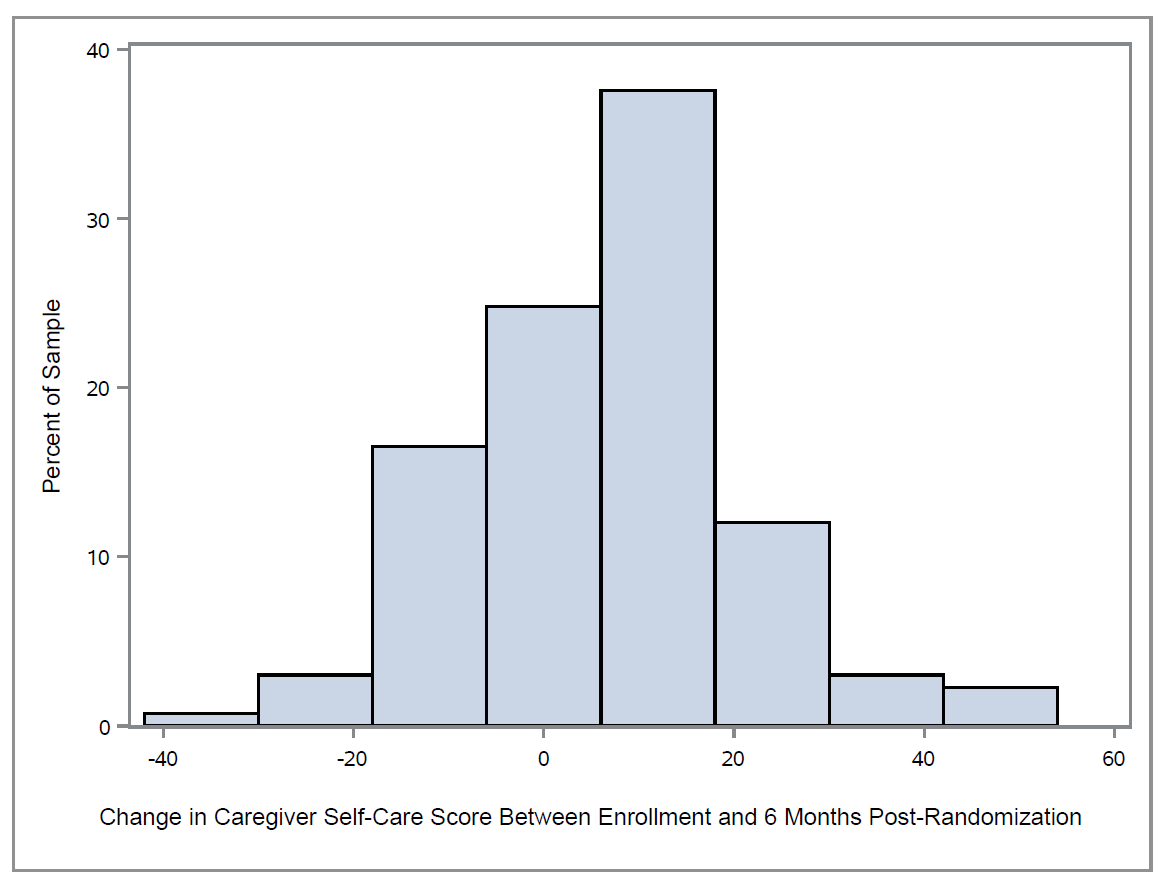


**Histograms Showing the Distributions of Patient Hospitalizations and Hospital Days by Caregiver Self-Care Improvement Category**

**
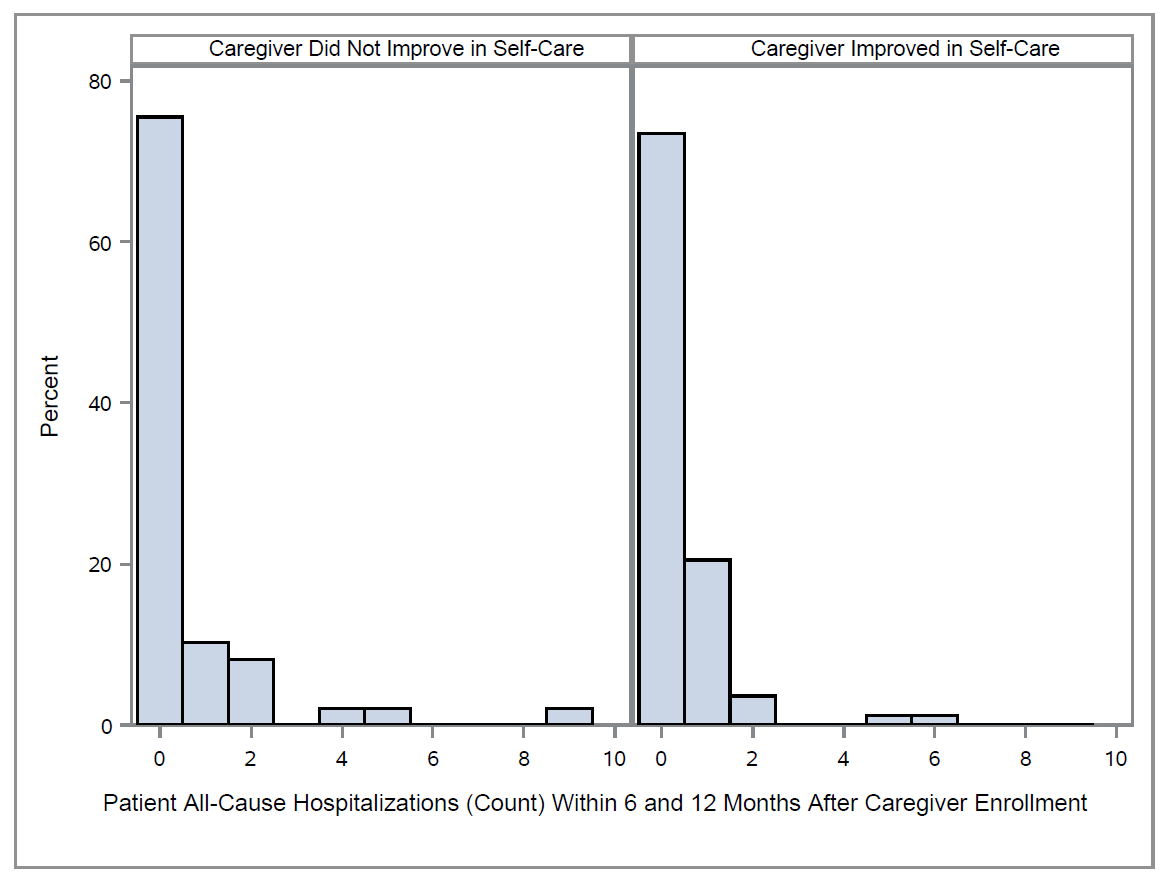
**  **
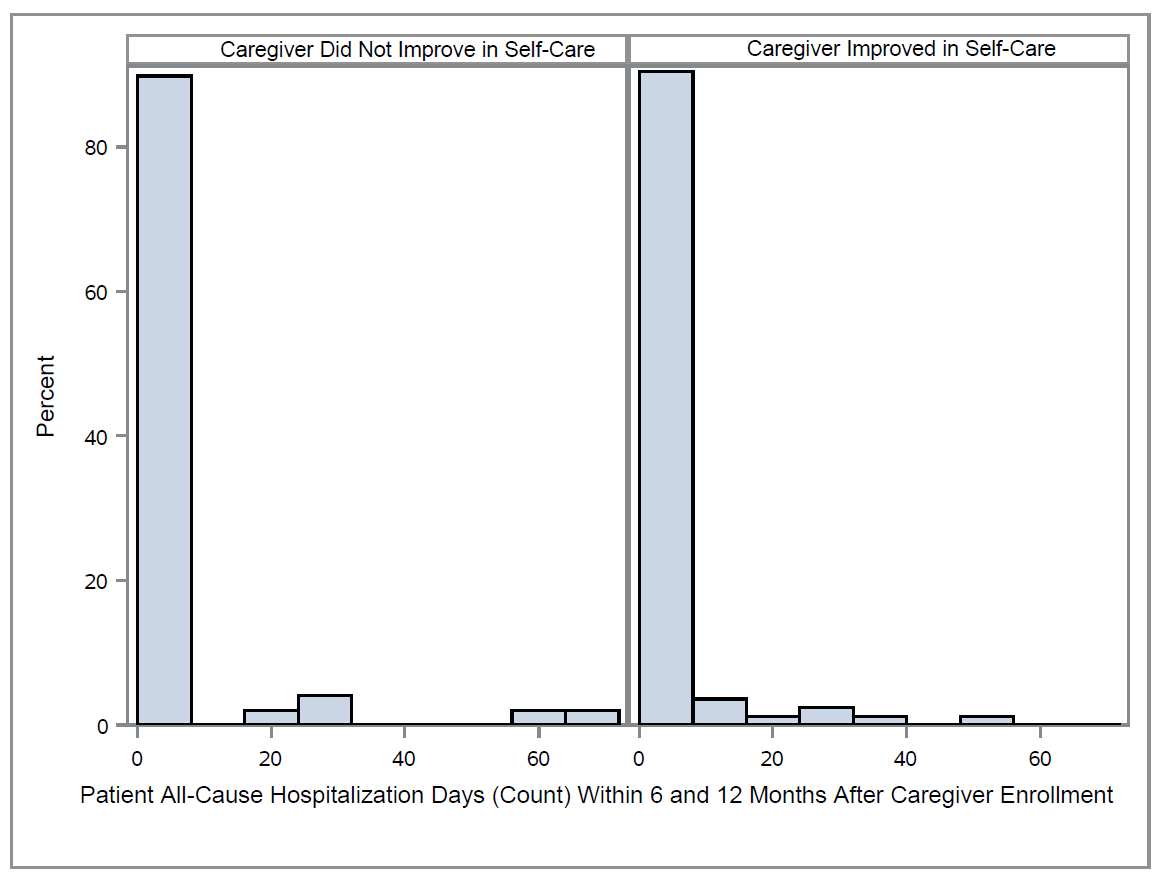
**
